# Correlation of quantitative background parenchymal enhancement with prognostic factors in breast cancer

**DOI:** 10.3389/fonc.2026.1761360

**Published:** 2026-08-19

**Authors:** Yuhua Liu, Weili Yu, Zhentao Wu, Yunxia Shen, Baohang Lin, Guan Huang, Zhihai Wu, Xiaoqin Yu

**Affiliations:** 1 Shenzhen Clinical Medical College, Guangzhou University of Chinese Medicine, Shenzhen, China; 2 Department of Radiology, Longgang District Maternity & Child Healthcare Hospital of Shenzhen City(Affiliated Shenzhen Women and Children’s Hospital (Longgang) of Shantou University Medical College), Shenzhen, China; 3 Department of Thyroid and Breast Surgery, Longgang Central Hospital of Shenzhen, Shenzhen, China; 4 Department of Pathology, Longgang Central Hospital of Shenzhen, Shenzhen, China; 5 Department of Oncology, Longgang Central Hospital of Shenzhen, Shenzhen, China; 6 Department of Ultrasound, Longgang Central Hospital of Shenzhen, Shenzhen, China

**Keywords:** background parenchymal enhancement, breast cancer, dynamic enhanced magnetic resonance imaging, Ki-67, prognostic factors

## Abstract

**Objectives:**

To semi-automatically quantify background parenchymal enhancement (BPE) in the contralateral healthy breast of patients with unilateral invasive breast cancer and investigate its correlation with prognostic factors, aiming to provide an imaging-based basis for breast cancer prognostic assessment.

**Materials and methods:**

A retrospective analysis was conducted on 124 patients with pathologically confirmed unilateral invasive breast cancer treated at Shenzhen Longgang Central Hospital between January 2019 and December 2024, BPE in the unaffected breast was quantified semi-automatically from DCE-MRI (phase 2 post-contrast) using 3D Slicer software (v5.6.2). Associations between quantitative BPE and prognostic factors (menopausal status, axillary lymph node metastasis, BMI, molecular subtype [Luminal A, Luminal B, HER-2+, triple-negative], ER, PR, HER-2, Ki-67) and systemic biomarkers (age, NLR, LMR, SII) were assessed using Mann-Whitney U, Kruskal-Wallis H, and Spearman’s correlation tests.

**Results:**

Quantitative BPE showed no significant differences based on menopausal status, axillary lymph node status, ER/PR/HER-2 status, BMI category, or molecular subtype (all *P* > 0.05). No significant correlations were found with age, NLR, LMR, or SII (all *P* > 0.05). Significantly lower BPE was observed in the Ki-67 high-expression group compared to the low-expression group (*P* = 0.002).

**Conclusion:**

Our study found that quantitative background parenchymal enhancement (BPE) was significantly lower in the Ki-67 high-expression group than in the low-expression group, suggesting a negative association with tumor proliferative activity. In addition, a certain correlation was also observed between BPE and the systemic inflammatory marker PLR. These findings provide preliminary clues for exploring BPE as an adjunctive imaging biomarker.

## Introduction

Breast cancer is the most commonly diagnosed cancer globally and the leading cause of cancer mortality in women worldwide (1). Its highly heterogeneous biology necessitates improved strategies for early prediction, treatment response assessment, and precision therapy. Background parenchymal enhancement (BPE), defined as the normal enhancement of fibroglandular tissue on dynamic contrast-enhanced magnetic resonance imaging (DCE-MRI), reflects underlying microvessel density and vascular perfusion (2). As an imaging marker potentially indicative of the treatment response, BPE holds promise for prognostic assessment in breast cancer. The typical manifestation of normal breast BPE is bilateral, symmetrical, and diffuse distribution. Given the symmetry between the two breasts, the healthy contralateral breast can serve as a reference for the normal glandular tissue of the affected side, and analysis of BPE in the contralateral breast of patients with unilateral invasive cancer provides a valuable reference, minimizing confounding factors from tumor effects, hormone fluctuations, or prior treatment (3). Previous studies have demonstrated that BPE reaches its peak approximately 210 seconds following contrast agent administration and subsequently stabilizes after 320 seconds, thus quantitative assessment is recommended during the early post-contrast phase of DCE-MRI for stability (4). Qualitative classification of BPE according to the latest version of the BI-RADS is the most commonly used method in current clinical practice (5), however, this method is unavoidably subjective and varies widely between researchers, making it difficult to standardize.

In recent years, standardized BPE quantification using dedicated software has gained traction, enabling reproducible measurements and direct correlation with other quantitative biomarkers. While fully automated segmentation offers superior accuracy, it demands complex algorithms and sophisticated programming. Semi-automated methods strike a practical balance—more accurate than qualitative assessment yet simpler to initialize and easier to refine (6, 7). Existing studies investigating correlations between qualitative BPE or quantitative BPE and established breast cancer prognostic factors including age, BMI, menopausal status, immunohistochemical markers and molecular subtypes, have yielded inconsistent findings (3, 4, 8–10).Furthermore, data exploring potential associations between quantitative BPE and axillary lymph node metastasis status or systemic immunoinflammatory indices remain limited. Therefore, this retrospective observational cohort analysis explored the correlation between BPE and prognostic factors of breast cancer by semi-automatic quantitative measurement of contralateral breast BPE in patients with unilateral invasive breast cancer, to provide a new basis for prognostic evaluation of breast cancer from the perspective of imaging.

## Materials and methods

This is a retrospective observational cohort study, which was reported in accordance with the STROBE guidelines for observational studies and approved by the Institutional Review Board (reference number: 2023ECPJ105).

### Study population

We retrospectively analyzed patients with unilateral invasive breast cancer who were treated at Longgang Central Hospital of Shenzhen from January 2019 to December 2024. All cases were confirmed by surgical pathology and had complete clinical, pathological and imaging data. The inclusion criteria were as follows: (1) unilateral invasive breast cancer confirmed via core needle biopsy, including cases with a carcinoma-*in-situ* component; (2) breast DCE-MRI performed within one week before treatment; (3) premenopausal patients underwent DCE-MRI during the second week of their menstrual cycle; (4) no history of biopsy, surgery, or any other treatment prior to the baseline DCE-MRI; (5) availability of complete hematologic test results. Exclusion criteria included: (1) incomplete clinical data; (2) missing or poor quality images unsuitable for analysis; (3) history of breast prosthesis implantation; (4) incomplete pathological results; (5) severe comorbidities including cardiac, hepatic, renal disorders, HIV, immune system diseases, nutritional disorders, or other malignancies; (6) symptoms of infection within one week prior to blood collection; (7) receipt of treatments that could affect hematological parameters within one week before the routine blood test.

**Figure 1:**
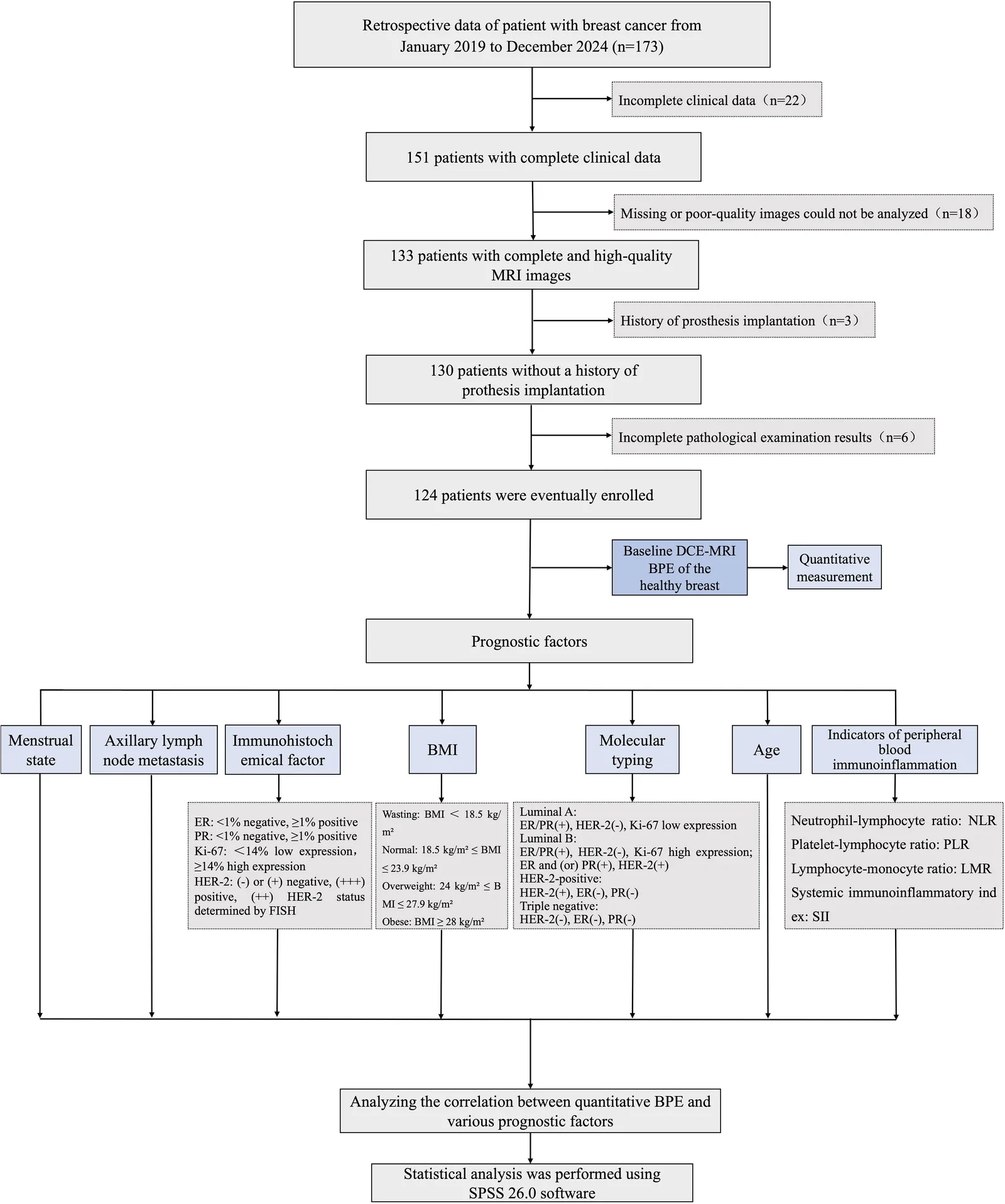
Flowchart of the study protocol.

According to the nadir criteria, a total of 124 female patients with unilateral invasive breast cancer were ultimately included as study subjects. This study is designed as a case series, and the technical workflow is shown in **Figure 1**.

### Image acquisition

All MRI examinations were performed on the 3 T MRI scanner (MAGNETOM Prisma, Siemens Healthcare, Erlangen, Germany) using a dedicated 16-channel breast coil. Patients were positioned in the prone position with both breasts placed naturally in the coil. Conventional and dynamic contrast-enhanced sequences were acquired with the following parameters:

Conventional sequences: axial T1 weighted imaging (T1WI): repetition time (TR) 6.0 ms, echo time (TE) 2.46 ms, field of view (FOV) 300 mm×300 mm, slice thickness 1.5 mm, matrix 326×384; axial T2 weighted imaging (T2WI) with fat suppression: TR 6460 ms, TE 71 ms, FOV 300 mm×300 mm, slice thickness 4.0 mm, matrix 326×384.

Dynamic contrast-enhancement sequence: TR 4.50 ms, TE 1.58 ms, FOV 300 mm×300 mm, slice thickness 1.5 mm, matrix 307×384. One pre-contrast phase was acquired prior to the intravenous administration of gadopentetate dimeglumine (Bayer Schering Pharma, Germany) at a dose of 0.2 mmol/kg body weight injected at a flow rate of 2.5 mL/s via a power injector, followed by a 20 mL saline flush at the same flow rate through an antecubital vein catheter. The second post-contrast phase was acquired at 60 seconds after contrast injection. A total of 9 dynamic phases were acquired, each with a duration of 30 seconds. An additional delayed phase was scanned 3 minutes after the start of the dynamic series.

Image preprocessing: No formal motion correction was applied, as all included cases were visually assessed to have minimal motion artifacts during the dynamic acquisition. However, we acknowledge this as a limitation, and cases with significant motion were excluded during quality control.

### BMI classification

Body mass index (BMI) was calculated as body mass in kilograms divided by the square of height in meters(kg/m^2^). According to the standards established by the Chinese Center for Disease Control and Prevention (CDC), BMI categories for Chinese adults are defined as follows: underweight (BMI <18.5 kg/m²), normal weight (18.5 kg/m² ≤ BMI ≤ 23.9 kg/m²), overweight (24 kg/m² ≤ BMI ≤ 27.9 kg/m²), and obese (BMI ≥ 28 kg/m²).

### Histopathological evaluation

All patients underwent either lumpectomy or mastectomy, and histopathological assessments were conducted on the resected specimens. Molecular receptor expression was evaluated via immunohistochemical staining. Lymph node metastasis (LNM) was confirmed through preoperative biopsy or surgical excision. In accordance with guideline criteria (10), estrogen receptor (ER) and progesterone receptor (PR) expression were considered negative if <1% of cells stained positive, and positive if ≥1%. Human epidermal growth factor receptor 2 (HER-2) results were interpreted follows: scores of 0 to 1+ were considered negative, 3+ was positive, and 2+ required further verification by fluorescence *in situ* hybridisation (FISH). Considering the principle of individualization and to enhance scientific rigor, this study based on the 2017 St. Gallen Expert Consensus (8), Ki-67 expression was classified as low if <14% of cells were positive, and high if ≥14%. Breast cancers were categorized into the following molecular subtypes: (1) Luminal A: ER and/or PR positive, HER-2 negative, and Ki-67 <14%; (2) Luminal B subdivided into Luminal B (HER-2 negative): ER and/or PR positive, HER-2 negative, and Ki-67 ≥14%; Luminal B (HER-2 positive): ER and/or PR positive, HER-2 positive; (3) HER-2 overexpression type: HER-2 positive, ER and PR negative; (4) Basal-like/Triple-negative type: ER, PR, HER-2 negative.

### Blood-based biomarkers

Pretreatment complete blood count data were retrieved from the hospital’s electronic medical records. The following parameters were collected: absolute neutrophil count (N), absolute lymphocyte count (L), absolute monocyte count (M), and absolute platelet count (P). The following ratios were computed: Neutrophil-to-lymphocyte ratio (NLR)=N/L; Platelet-to-lymphocyte ratio (PLR)=P/L; Lymphocyte-to-monocyte ratio (LMR)=L/M; Systemic immune-inflammation index (SII)=N×P/L.

### Quantitative analysis of BPE

**Figure 2:**
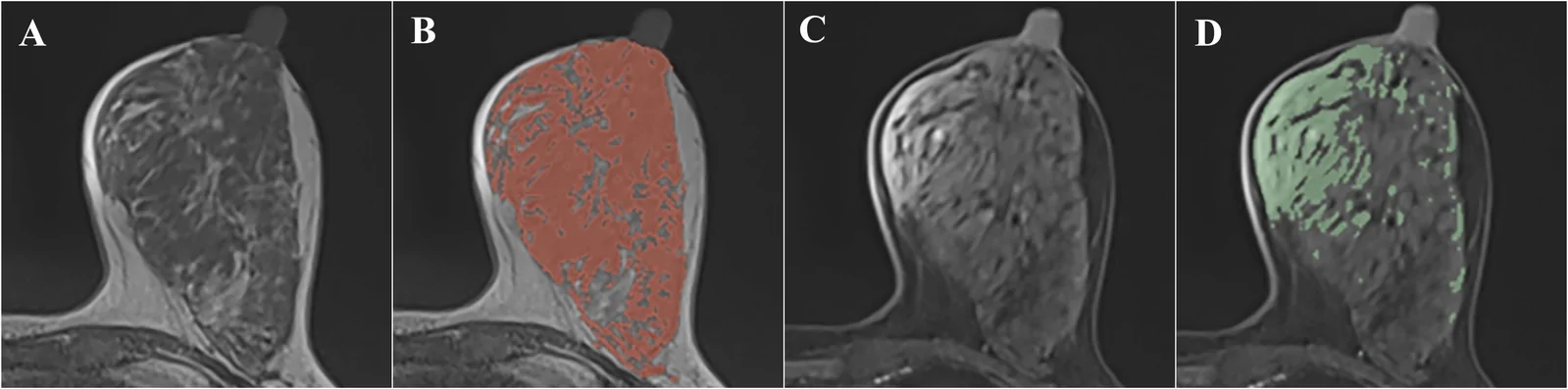
The proportion of background parenchymal enhancement of the patient was 42.9%. **(A)** Axial T1-weighted precontrast image with the contralateral breast selected as the region of interest. **(B)** Segmentation of fibroglandular tissue (highlighted in red) on the T1-weighted image. **(C)** Early postcontrast phase (second dynamic phase) image of the contralateral breast. **(D)** Segmentation of enhancing BPE (highlighted in green) on the early postcontrast image.

Quantitative assessment of background parenchymal enhancement (BPE) was performed using 3D Slicer software (version 5.6.2; https://www.slicer.org). The contralateral breast of patients with unilateral invasive breast cancer was analyzed on baseline DCE-MRI. After importing the images, the precontrast T1-weighted sequence was selected. A threshold mask was applied to isolate fibroglandular tissue (FGT), and the volume brush tool was used to segment FGT from adipose tissue. The software automatically computed the FGT volume. Next, the early postcontrast phase (second phase) was selected. Using the same thresholding and volume brush technique, enhancing glandular tissue was segmented, and the BPE volume was automatically calculated (11). Quantitative BPE was expressed as a percentage: BPE% = [BPE volume/FGT volume]×100%. A schematic of this process is illustrated in **Figure 2**. The quantitative analysis of BPE was independently performed by three senior radiologists specializing in breast imaging. In cases of discordant interpretation, consensus was reached through consultation.

### Statistical analysis

All statistical analyses were performed using SPSS software (version 26.0, IBM SPSS, Chicago, IL, USA). The normality of quantitative data was assessed using the Kolmogorov-Smirnov test. Normally distributed data are expressed as mean ± standard deviation (x̄ ± s) and were compared using the student’s t-test for equal variances or Welch’s t-test (t’-test) for unequal variances. Non-normally distributed data are presented as median (interquartile range) and compared using the Mann-Whitney U test for two-group comparisons or the Kruskal-Wallis H test for multi-group comparisons. Correlation analyses were conducted based on the distribution of the data: Pearson correlation was used if both datasets were normally distributed, and Spearman correlation was applied if one or both datasets deviated from normality. A two-sided p-value < 0.05 was considered statistically significant.

## Results

### Baseline characteristics

The clinical and pathologic characteristics of the patients are summarized in **Table 1**. A total of 124 female patients were included in the study with a mean age of 46.14 ± 9.32 years (range: 24–71 years old). Molecular subtyping classified the cases as follows: Luminal A (n = 18), Luminal B (n = 70), HER-2 overexpression (n = 20), and triple-negative (n = 16). Receptor status analysis revealed ER positivity in 86 cases (86/124, 69.4%), PR positivity in 80 cases (80/124, 64.5%), and HER-2 positivity in 41 cases (41/124, 33.1%). High Ki-67 expression (≥14%) was observed in 84 patients (84/124, 67.7%). Based on BMI categories, 3 patients (2.4%) were underweight, 62 (50.0%) were normal weight, 41 (33.1%) were overweight, and 18 (14.5%) were obese. Regarding menopausal status, 96 (77.4%) patients were premenopausal and 28 (22.6%) were postmenopausal. Axillary lymph node metastasis was present in 80 (64.5%) patients and absent in 44 (35.5%), as shown in **Table 1**.

**Table 1:** Clinical and pathological data of breast cancer patients.

Characteristics	
Clinical characteristics	
Mean age (years)	46.14 ± 9.32
BMI (kg/m2, %)	
Wasting	3(2.42)
Normal	62(50.00)
Overweight	41(33.06)
Obese	18(14.52)
Menopausal status (%)	
Premenopausal	96(77.42)
Postmenopausal	28(22.58)
Location of lesion (%)	
Upper outer quadrant	38(30.65)
Lower outer quadrant	8(6.45)
Upper inner quadrant	11(8.87)
Lower inner quadrant	6(4.84)
Cross quadrant	61(49.19)
Pathological characteristics	
Axillary lymph node metastasis (%)	
No	44(35.48)
Yes	80(64.52)
ER (%)	
Negative	38(30.65)
Positive	86(69.35)
PR (%)	
Negative	44(35.48)
Positive	80(64.52)
HER-2 (%)	
Negative	83(66.94)
Positive	41(33.06)
Ki-67 (%)	
Low expression	40(32.26)
High expression	84(67.74)
Molecular typing (%)	
Luminal A	18(14.52)
Luminal B	70(56.45)
HER-2-positive	20(16.13)
Triple negative	16(12.90)

BMI, Body Mass Index; ER, estrogen receptor; PR, progesterone receptor; HER-2, human epidermal growth factor receptor 2; Ki-67, Cell proliferation index.

There was no statistically significant difference (P>0.05) in quantitative BPE between premenopausal and postmenopausal groups, axillary lymph node non-metastatic and metastatic groups, and ER, PR, HER-2 negative and positive groups. There was no significant correlation (P>0.05) between quantitative BPE and patient age, peripheral blood immune inflammation NLR, LMR, SII. There was a statistically significant difference (P<0.05) between Ki-67 low expression group and high expression group, high PLR group and low PLR group, as shown in **Table 2**.

**Table 2:** Association of quantitative BPE with clinicopathological characteristics in breast cancer patients.

Prognostic factors	BPE%	*Z*	*P value*
Age/years			
≤46.14(n=69)	28.20(30.12)	-1.732	0.085
>46.14(n=55)	11.33(30.12)		
Menopausal status			
Premenopausal(n=96)	24.00(46.48)	-1.243	0.214
Postmenopausal(n=28)	11.08(27.03)		
Axillary lymph node metastasis			
No(n=44)	18.80(37.01)	-0.470	0.638
Yes(n=80)	24.00(44.44)		
Immunohistochemical factor			
ER negative(n=38)	21.66(45.39)	-0.363	0.717
ER positive(n=86)	18.19(38.14)		
PR negative(n=44)	21.66(43.47)	-0.564	0.573
PR positive(n=80)	18.19(39.85)		
HER-2 negative(n=83)	18.86(44.95)	-0.438	0.661
HER-2 positive(n=41)	24.08(35.61)		
Ki-67 Low expression(n=40)	36.82(37.76)	-3.079	**0.002**
Ki-67 High expression (n=84)	11.57(25.36)		
Peripheral blood immunoinflammatory biomarkers			
NLR			
≤2.30(n=62)	19.07(31.62)	-0.840	0.401
>2.30(n=62)	21.47(47.26)		
PLR			
≤158.98(n=62)	11.54(30.97)	-2.039	**0.041**
>158.98(n=62)	28.57(45.82)		
LMR			
≤4.26(n=62)	23.31(46.10)	-0.425	0.671
>4.26(n=62)	18.13(30.16)		
SII			
≤631.97(n=62)	13.98(31.08)	-1.874	0.061
>631.97(n=62)	26.26(46.32)		

Differences in quantitative BPE between stratified BMI, location of lesion and molecular types.Bolded values mean p < 0.05.

No statistically significant differences in quantitative background parenchymal enhancement (BPE) were observed when patients were stratified by BMI categories, location of lesion or molecular subtypes (all *P* > 0.05), as detailed in **Table 3**.

**Table 3:** Analysis of quantitative BPE across BMI categories, location of lesion and molecular subtypes.

Prognostic factors	BPE%	*Z*	*P-*Value
BMI			
Wasting	19.39(-)	3	0.731
Normal	16.37(41.93)		
Overweight	21.16(32.11)		
Obese	27.02(47.57)		
Location of lesion			
Upper outer quadrant(n=38)	22.62(41.00)	4	0.066
Lower outer quadrant(n=8)	10.70(20.65)		
Upper inner quadrant(n=11)	8.52(18.76)		
Lower inner quadrant(n=6)	44.37(49.18)		
Cross quadrant(n=61)	22.78(49.94)		
Molecular subtypes			
Luminal A	33.90(35.76)	3	0.282
Luminal B	14.26(32.97)		
HER-2-positive	21.20(46.52)		
Triple negative	21.33(50.96)		

### Multivariable logistic regression

**Figure 3:**
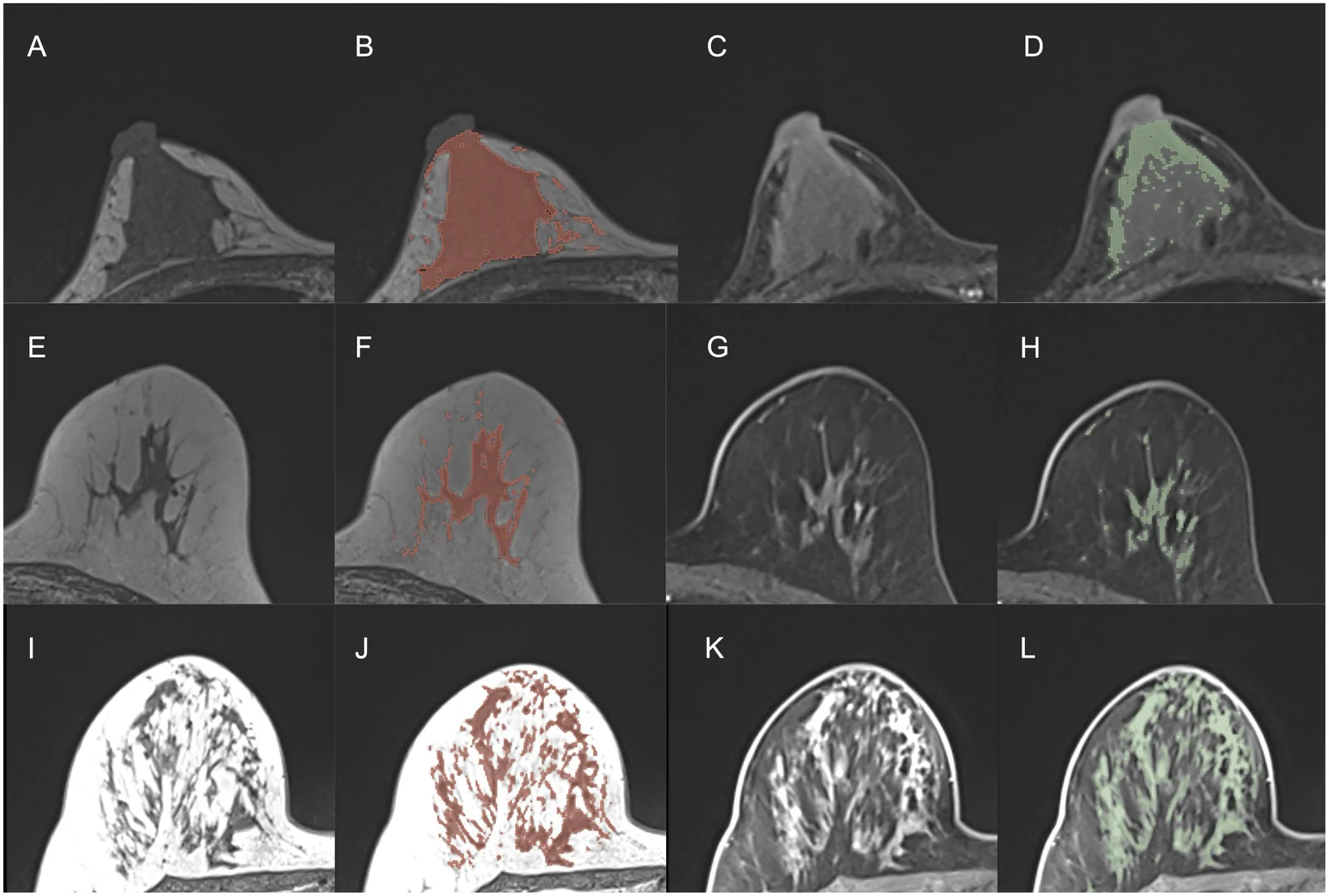
The proportion of background parenchymal enhancement of the patients were 4.1%, 10.5% and 69.1%. **(A, E, I)** Axial T1-weighted precontrast image with the contralateral breast selected as the region of interest. **(B, F, J)** Segmentation of fibroglandular tissue (highlighted in red) on the T1-weighted image. **(C, G, K)** Early postcontrast phase (second dynamic phase) image of the contralateral breast. **(D, H, L)** Segmentation of enhancing BPE (highlighted in green) on the early postcontrast image.

Different proportion of background parenchymal enhancement are shown in **Figure 3**. Multivariable logistic regression analysis, with high Ki-67 expression as the outcome, revealed that BPE% was the independent predictor (**Table 4**). Lower BPE% was significantly associated with increased likelihood of high Ki-67 expression (OR = 0.981, 95% CI: 0.966–0.996, P = 0.014). These findings confirm that the inverse relationship between quantitative BPE and Ki-67 expression is independent of hormone receptor status, HER-2, menopausal status, and BMI.

**Table 4:** Multivariable logistic regression analysis of factors associated with high Ki-67 expression.

	Multivariate analysis		
	OR	95%CI	P
BPE%	0.981	0.966-0.996	**0.014**
ER	0.554	0.101-3.024	0.495
PR	1.054	0.189-5.887	0.951
HER-2	0.878	0.349-2.209	0.782
Menopausal status	2.072	0.660-6.502	0.211
BMI (kg/m2)	1.026	0.914-1.152	0.661

Bolded values mean p < 0.05.

## Discussion

In this study, semi-automated quantitative BPE measurement of the contralateral breast was performed using 3D Slicer on conventional T1WI and second-phase post-contrast MRI in patients with unilateral invasive breast cancer, to investigate its correlation with prognostic factors. Quantitative BPE was significantly associated with Ki-67 expression status and PLR, whereas no significant correlations were found with other prognostic factors.

BPE is defined as the normal enhancement of glands after injection of gadolinium contrast agent, which is related to the microvascular density and vascular perfusion of breast tissue. As BPE is influenced by hormonal fluctuations, pathological alterations, and clinical treatment effects, prior evidence (3) has established that the contralateral healthy breast may serve as a valid surrogate for normal glandular tissue of the ipsilateral breast given inherent bilateral symmetry. Therefore, BPE analysis was performed on the contralateral breast in this study. However, due to the wide categorical intervals in conventional qualitative grading scales and subjective differences, it is difficult to achieve standardization. In recent years, the use of quantitative analysis software for standardized measurement of BPE has gradually become a trend. With the help of automated measurement technology, personalized measurement and evaluation of BPE enable reproducible assessment and facilitate correlation with other quantitative indicators. Therefore, this study aimed to investigate the correlation between quantitative BPE and prognostic factors in invasive breast cancer.

Our results revealed that quantitative BPE was lower in tumors with high Ki-67 expression compared to those with low Ki-67 expression, suggesting a possible inverse relationship between BPE and tumor proliferative activity. Ki-67 is a nuclear non-histone protein that serves as a proliferation marker in cell populations, and is widely recognized as one of the most reliable indicators for assessing cell proliferation in malignant tumors (12). Previous studies have consistently linked elevated Ki-67 expression to more aggressive tumor biology and poorer clinical outcomes in breast cancer. Javaid et al. (13) reported that high Ki-67 levels were associated with higher tumor grade and worse prognosis. Similarly, Sandilya et al. (14) observed significant correlations between Ki-67 expression and clinicopathological features such as tumor size and histological grade. Furthermore, Giridhar et al. (15) demonstrated that a high Ki-67 index was not only associated with higher tumor grade and advanced pathological stage but also correlated with unfavorable molecular subtypes and negative hormone receptor status (ER and PR), reinforcing the value of Ki-67 as a marker of biologically aggressive behavior and poor prognosis. The underlying mechanisms may involve abnormalities in tumor vasculature, such as increased permeability, tortuosity, dilation, and aberrant branching, as well as morphological changes in endothelial cells (16). These alterations contribute to elevated vascular resistance, reduced blood flow velocity, and heterogeneous perfusion, ultimately leading to a hypoxic and acidotic tumor microenvironment (TME). Hypoperfused hypoxic regions and leakage of plasma components further increase interstitial pressure, which compresses blood vessels and impedes perfusion. The proliferative and invasive activity of tumor cells exacerbates these pathophysiological conditions, causing damage to surrounding normal tissues and further reducing regional blood flow (17). Therefore, high Ki-67 expression may drive these pathophysiological alterations, damaging adjacent normal breast parenchyma and reducing regional perfusion, ultimately manifesting as decreased BPE. In addition, inadequate tumor vasculature often fails to support rapid tumor growth. Hypoxia stimulates both tumor and stromal cells to produce excess extracellular matrix components that promote fibrosis. The fibrotic response in turn compresses blood vessels, leading to their collapse (18).

Given that BPE is influenced by angiogenesis and inflammatory processes (3), the observed reduction in BPE in cases with high Ki-67 expression may reflect these vascular abnormalities and their functional consequences. The relationship between BPE and Ki-67 remains controversial. Mao et al. and Liu et al. (19, 20), after analyzing the correlation between BPE and immunohistochemical factors, showed no significant difference in BPE between high and low Ki-67 expression groups, which was inconsistent with the results of this study. This discrepancy may be attributed to variations in Ki-67 cutoff values across studies. For instance, considering the principle of individualization and to enhance scientific rigor, the St. Gallen Expert Consensus changed the Ki-67 threshold from 20% in 2013 to 14% in 2017 (21). Moreover, differences in BPE assessment methods—such as qualitative versus quantitative approaches—as well as variations in MRI equipment, analysis software, sample sizes, and cohort characteristics may also contribute to inconsistent findings. Therefore, further standardized investigations are warranted to clarify the association between BPE and Ki-67.

ER, PR, and HER-2 are established independent prognostic predictors in breast cancer and remain the most widely used biomarkers for guiding treatment decisions in clinical practice. However, the association between BPE and these immunohistochemical factors remains controversial (22). In the present study, no statistically significant difference in BPE was observed between ER-, PR-, and HER-2-negative and positive groups, consistent with findings reported by domestic researchers (11). Our results also align with prior studies in certain respects. For instance, Li et al. (23) conducted a retrospective study of 163 patients with unilateral invasive breast cancer and reported that qualitative BPE was positively correlated with ER positivity, but showed no significant association with PR or HER-2 status. Subsequently, domestic scholars (11) investigated the relationship between BPE and tumor biomarkers using both qualitative and quantitative approaches, finding that qualitative BPE differed significantly between HER-2-negative and positive groups, whereas neither qualitative nor quantitative BPE differed significantly across ER or PR expression statuses. Liu et al. (19) quantitatively analyzed BPE in 56 patients with unilateral breast cancer and found that the proportion of high BPE distribution was higher in HER-2-positive tumors than in the HER-2-negative group, with statistical significance. However, no significant correlations were observed with ER or PR. Collectively, the relationship between BPE and immunohistochemical factors remains elusive, and further large-scale studies are warranted to clarify this issue.

In recent years, Peripheral immunoinflammatory biomarkers indices NLR, PLR, LMR and SII, derived from the ratios of inflammatory cells, have been used as effective parameters reflecting systemic immune-inflammatory responses due to their greater stability compared to individual blood cell counts. Savioli et al. (24) investigated the prognostic value of circulating biomarkers of systemic inflammatory response in primary operable breast cancer, demonstrating that elevated NLR was closely associated with shorter OS, DFS, and breast cancer-specific survival (BCSS). Song et al. (25) examined the prognostic significance of pretreatment LMR in ER-positive/HER2-negative patients, with multivariate analysis revealing that low LMR was associated with unfavorable outcomes. Additionally, one study (26) analyzing NLR, PLR, and LMR in 808 breast cancer patients who underwent neoadjuvant chemotherapy (NAC) followed by surgery identified LMR as the sole independent predictor of NAC efficacy among these inflammatory markers. Another study (27) demonstrated through 5-year follow-up that elevated SII was a risk factor for breast cancer recurrence and metastasis, suggesting that increased SII often indicates poor disease prognosis. Numerous investigators have since explored the value of SII in predicting breast cancer prognosis and treatment response, with findings indicating that SII serves as an independent predictor of pathological complete response to neoadjuvant chemotherapy and outperforms both NLR and PLR (28). Although our data suggested an inverse correlation between BPE and NLR, and LMR and SII, these relationships did not reach statistical significance.

Gong et al. (29) evaluated the correlation between PLR and breast cancer prognosis as well as clinicopathological features in 7484 breast cancer patients. The results showed that high PLR was associated with tumor stage and lymph node metastasis. In addition, many scholars have reached consistent conclusions through analysis, pointing out that higher PLR indicates poor prognosis and is associated with shorter OS and DFS in breast cancer patients (24, 30). On this basis, Ouissam et al. (31) studied PLR in a group of patients with non-metastatic inflammatory breast cancer and found that the 5-year DFS and OS of patients in the low PLR group were significantly better than those in the high PLR group. Subsequently, Qi et al. (32) explored the prognostic value of PLR in breast cancer patients receiving NAC, demonstrating that elevated PLR was significantly associated with lower pCR rates and inferior OS and DFS. PLR is defined as the ratio of platelets to lymphocytes. Tumor cells can activate platelets, and activated platelets directly adhere to tumor cells, facilitating immune evasion. Simultaneously, activated platelets promote tumor angiogenesis and stromal formation through the secretion of vascular endothelial growth factor (VEGF) and the recruitment of inflammatory cells, thereby fostering tumor proliferation and progression (33). Lymphocytes can secrete cytokines to activate anti-tumor immunity and directly kill tumor cells, thereby inhibiting tumor cell proliferation and metastasis. Their infiltration into the breast cancer microenvironment reflects the level of host adaptive immune response and is recognized as a hallmark of treatment sensitivity and favorable prognosis in breast cancer (34). Therefore, high PLR often indicates a “pro-tumorigenic” state in patients. However, no studies have yet investigated the correlation between BPE and peripheral blood immunoinflammatory indices. BPE is related to the microvessel density and vascular perfusion of breast tissue. Higher BPE usually indicates that the glandular tissue has abundant blood flow and a pro-angiogenic environment, which is conducive to the proliferation and metastasis of tumor cells. In our study, the difference in quantitative BPE between the high PLR group and the low PLR group was statistically significant. This may suggest that high BPE areas represent immunosuppressive and pro-inflammatory microenvironments, which suppresses anti-tumor immunity and instead facilitates tumor immune escape.

Previous studies (35, 36) have demonstrated a significant association between qualitative BPE and menopausal status, with postmenopausal women exhibiting lower BPE than premenopausal women. In the present study, semiautomated quantitative analysis revealed no statistically significant difference in BPE between premenopausal and postmenopausal groups, likely attributable to limited sample size and imbalanced group distribution. Nevertheless, a trend toward lower BPE in postmenopausal patients was visually apparent.

Axillary lymph-node metastasis is a critical prognostic factor in breast cancer, guiding risk stratification and treatment decisions. Zhang et al. (37) analyzed the correlation between BPE and lymphovascular invasion (LVI), demonstrating a significant difference in BPE between the LVI-positive and LVI-negative groups. Given that LVI is closely associated with axillary lymph node metastasis in breast cancer patients, it is hypothesized that a certain correlation exists between BPE and axillary lymph node metastasis. Similar to the findings of Choi et al. (38), we observed no significant difference in qualitative BPE between node-positive and node-negative patients. To our knowledge, no prior studies have investigated the correlations between quantitative BPE and axillary lymph-node metastasis or peripheral immunoinflammatory biomarkers. Therefore, further research is warranted to elucidate these relationships.

Our findings showed no statistically significant difference in BPE among molecular subtypes, consistent with the results reported by Wu et al. and Mao et al. (20, 35) Further research is warranted to clarify this association.

BMI, a widely used indicator of adiposity and health status, has been increasingly recognized as a factor associated with breast cancer risk and long-term prognosis (39). Brown et al. (40) demonstrated a correlation between absolute BPE (measured in cm²) and BMI in premenopausal women at high risk for breast cancer. Similarly, Brooks et al. (41) observed a positive association between BMI and BPE in 415 women without breast cancer, reporting that each 5-unit increase in BMI was associated with a 14% and 44% increase in high BPE among premenopausal and postmenopausal women, respectively. In contrast, our study found no significant difference in quantitative BPE across BMI strata, although the quantitative approach employed may have reduced inter-reader variability.

We observed a negative correlation between quantitative BPE and patient age, though this did not reach statistical significance. This trend suggests that higher BPE may be more prevalent in younger patients, a finding supported by Wu et al, Hellgren et al, and Sallam et al. (35, 36, 42) BPE is a dynamic process that varies among individuals and within the same individual over time. The progressive decline in BPE with age likely reflects physiological changes in breast tissue composition: younger women typically have denser breast tissue with greater vascularity, resulting in more pronounced contrast uptake and enhancement, whereas advancing age is accompanied by progressive fatty replacement and reduced vascular density, leading to decreased BPE levels.

In summary, our study identified a statistically significant difference in quantitative BPE between patients with high versus low Ki-67 expression, as well as between the low- and high-PLR groups, but found no significant correlations with age, menopausal status, axillary lymph node metastasis, immunohistochemical factors (ER, PR, HER-2), molecular subtypes, BMI, or peripheral immunoinflammatory biomarkers (NLR, LMR, SII). Nevertheless, these results do not preclude the potential value of BPE in prognostic evaluation of breast cancer.

Several limitations should be acknowledged. This was a single-center retrospective study with a limited sample size, which may affect the generalizability of our findings. In addition, subgroup analyses were constrained by uneven sample distribution. The use of semi-automated software limited BPE quantification to the contralateral breast only. Furthermore, reliability assessment was not pre-specified, precluding retrospective ICC calculation and future studies will prospectively archive segmentation data to report formal reliability metrics. Finally, this study lacked survival or treatment outcome data for the included breast cancer patients. Therefore, further prospective studies with long-term follow-up are warranted to establish definitive clinical conclusions. At last, the relationship between BPE and Ki-67 is exploratory and hypothesis-generating, and the observed associations do not imply causality. Therefore, any mechanistic interpretations are speculative and require validation through prospective or experimental studies.

## Conclusion

Our study found that quantitative background parenchymal enhancement (BPE) was significantly lower in the Ki-67 high-expression group than in the low-expression group, suggesting a negative association with tumor proliferative activity. In addition, a certain correlation was also observed between BPE and the systemic inflammatory marker PLR. These findings provide preliminary clues for exploring BPE as an adjunctive imaging biomarker.

## Data Availability

The raw data supporting the conclusions of this article will be made available by the authors, without undue reservation.
